# Outcome of adult congenital heart disease patients undergoing cardiac surgery: clinical experience of dr. Sardjito hospital

**DOI:** 10.1186/s12919-019-0178-5

**Published:** 2019-12-16

**Authors:** Juni Kurniawaty, Yunita Widyastuti

**Affiliations:** Department of Anesthesiology and Intensive Care, Faculty of Medicine, Public Health, and Nursing, Universitas Gadjah Mada, Yogyakarta, Indonesia

**Keywords:** Adult congenital heart disease, Mortality, Hospital length of stay, ICU length of stay, Risk factors

## Abstract

**Background:**

Patients with congenital heart disease require surgery to correct the cardiac defect they had in order to prevent heart failure. Unfortunately, data regarding outcome of adult CHD in Indonesia is still limited. In contrast with developed countries, many congenital heart surgery patients in developing countries are adults. The purpose of this study was to investigate the outcomes of cardiac surgery procedures among adult congenital heart disease patients, and what factors that might influence the outcome of surgery.

**Methods:**

A retrospective study was performed on adult congenital heart disease patients undergoing cardiac surgery at Dr. Sardjito Hospital between April 2018 and March 2019. Variables included in the study were demographic characteristics, laboratory test results, comorbidities, premedication, Cardiopulmonary Bypass (CPB) and ischemia duration were included in the analysis. Outcomes were in-hospital mortality, hospital length of stay, and ICU length of stay.

**Results:**

A total of 25 congenital heart disease patients [19 Atrial Septeal Defect (ASD) patients, and 6 Ventricular Septal Defect (VSD) patients] underwent a cardiac surgery procedure at Dr. Sardjito Hospital during the study period. Mean age was 31 ± 14.92 years. The majority of patients had pulmonary hypertension. During the study period, none of the patients died during postoperative care in the hospital, mean hospital length of stay (LOS) was 8.35 ± 3.39 days and ICU LOS was 26.53 ± 11.33 h.

**Conclusion:**

Surgery in adult patients with congenital heart disease may be successfully performed with low morbidity and mortality.

## Background

Congenital heart disease (CHD) is defined as a structural or functional cardiovascular abnormality that develops at birth, and is still considered as one of the most common birth defects, with a prevalence of 5–8 per 1000 live births in Western countries [1]. With rapid progress in diagnosis and management, most children born with CHD are expected to have a normal and productive life. Among various factors, identification of CHD and timely intervention are crucial for the outcome of children with CHD. Antenatal CHD detection is now considered a standard care in developed countries, thus improving the final outcome. However, in most countries with low and moderate income, this facility is still limited, and majority of patients from developing countries are not diagnosed early. Delays in diagnosis may lead to suboptimal management and poor outcome since complications may have already develop. Comorbidities, especially malnutrition, may affect therapeutic intervention [2]. The purpose of this study was to investigate the outcomes of cardiac surgery procedures among adult congenital heart disease patients, and what factors that might influence the success of the procedures.

## Methods

A retrospective study was performed on adult patients undergoing cardiac surgery due to congenital heart disease in Dr. Sardjito Hospital, Yogyakarta, Indonesia between April 1, 2018 and March 31, 2019 and recorded in Indonesia Cardiovascular Anesthesia Registry Database (INACARD) database. Adult patients with a diagnosis of congenital heart disease who underwent cardiac surgery were included in the study. Subjects under 18 years of age were excluded from the study. All data recorded in INACARD was prospectively collected and each report was reviewed by a senior anesthesiologist.

The following parameters were included in the study: demographic characteristics, laboratory test results, comorbidities, premedications, Cardiopulmonary Bypass (CPB) duration and ischemia duration. Outcomes were in-hospital mortality, hospital legth of stay (LOS), and Intensive Care Unit (ICU) LOS. In-hospital mortatily was defined as all-cause mortality events in CHD patients during post-operative period. Forty eight hours was used as cutoff point to consider prolonged ICU LOS. Hospital length of stay was measured between the day of surgery and the day of hospital discharge. Hospital LOS of 7 days or more was considered as prolonged hospital LOS. The collected data was assessed regarding demographic characteristics, laboratory and intraoperative data, in-hospital mortality, hospital LOS, and ICU LOS. If possible, further analysis would be performed to investigate the potential factors that affected outcomes. Univariate analysis were performed using chi square test or Fisher exact test for categorical variables and Student’s t test or Mann Whitney U test for continuous variables. Multivariate analysis with logistic regression would be performed for all variables with significant association in bivariate analysis. Results were considered as significant if *p* value < 0.05 with a 95% confidence interval. Statistical analysis was performed using STATA version 13 software (Stata Corp, Texas, USA).

## Results

Forty-nine patients underwent cardiac surgery in Dr. Sardjito hospital during the study period. Of these, 25 patients had congenital heart disease. No patients died during hospitalization. Mean post-operative hospital length of stay was 8.35 ± 3.39 days. All patients were treated in ICU for less than 2 days (Table 1).

**Table 1:** Demographic characteristics of congenital cardiac surgery patients

Variables	n (Percentage) or Mean ±SD
Gender	
Male	5(20)
Female	20(80)
Age	31.15 ± 13.63
Weight (kg)	42.8 ± 12.92
Height (cm)	150.13 ± 11.9
Post-op length of stay (days)	8.35 ± 3.39
CPB time (min)	56.00 ± 32.17
Ischemic time (min)	30.70 ± 27.80
congenital cardiac lesion type	
ASD	19(76)
VSD	6(24)
others	0
Smoking history	0
Alcohol history	0
Previous cardiac surgery	0
Allergy history	1(4)
Diabetic history	0
Dyslipidemia history	0
Renal failure history	0
Hypertension history	1(4)
Cerebrovascular disease history	1(4)
Chronical lung disease history	0

The most common lesion found in our patients were Atrial Septal Defect (ASD), reported in 19 patients (76%) and followed by Vetricular Septal Defect (VSD) in 6 patients (24%). Mean age of patients was 31.00 ± 14,92 years. Most patients were women (20 patients, 80%), and only 5 patients (20%) were men. No patients reported history of smoking, alcohol, previous cardiac surgery, diabetes, dyslipidemia, and cerebrovascular diseases.

The result of preoperative evaluation (Table 2) found that no patients had angina, cyanosis, carotid occlusion, and carotid stenosis. Four patients had chronic heart failure. Active endocarditis were reported in 2 patients. Two patients had arrhythmia. Most patients in the study had pulmonary hypertension (15 patients, 60%). Regarding Cardiac Anesthesia Risk Evaluation (CARE) score, 9 patients (36%), 5 patients (20%), and 11 patients (44%) had CARE scores 1, 2, and 3, respectively. Most patients had New York Heart Association (NYHA) scale I and II (10 and 12 patients, respectively), and only 3 patients (12%) were classified as NYHA III. No patients had NYHA IV.

**Table 2:** Pre-operative evaluation results of congenital cardiac surgery patients

Variables	N (Percentage) or Mean ±SD
Angina	0
Chronic heart failure	4(16)
Cyanosis	0
Active endocarditis	2(8)
Arrhythmia	2(8)
Claudication	0
Aortic occlusion	0
Carotid stenosis > 50%	0
CARE score	
1	9(36)
2	5(20)
3	11(44)
NYHA	
I	10(40)
II	12(48)
III	3(12)
IV	0
Pulmonary hypertension	15(60)
Preoperative Medication	
Beta blocker	9(36)
ACE inhibitor	4(16)
ARB	2(8)
Nitrate	0
steroid	0
aspirin	1(4)
simvastatin	0
Anti-hypertensive drugs	6(24)
Inotropic	2(8)
Diuretic	5(20)
Anticoagulant	4(16)
Mean Arterial Pressure	79.71 ± 7.15
Haemoglobin	13.12 ± 1.50
Hematocrit	39.41 ± 3.50
White Blood Count	7.30 ± 2.34
Platelet	286.16 ± 83.13
PaCO_2_	33.65 ± 11.03
PaO_2_	85.55 ± 14.87
HCO_3_	21.65 ± 4.05
BE	−1.88 ± 4.14
SaO_2_	97.22 ± 1.59

Analysis of the medication history found that beta-blockers were the most prescribed drugs in patients with congenital heart disease, where 9 patients (36%) in the study were treated with beta-blocker. A total of 6 patients (24%) received anti-hypertensive drugs. Diuretic drug were administered in 5 patients.

Since all patients with adult congenital heart disease who underwent cardiac surgery in our study were alive during post-operative hospitalization, we did not perform further multivariate analysis for in-hospital mortality. Regarding hospital LOS, 11 patients had prolonged hospital LOS. Univariable analysis for hospital LOS found that age, gender, NYHA class, CARE score, and PH did not have significant association with hospital LOS (Table 3).

**Table 3:** Univariable analysis for hospital LOS

Variables	Hospital LOS		*P* value (Fisher’s exact test)
	≤ 7 days (14 subjects)	> 7 days (11 subjects)	
Gender			
Male	1 (7.1%)	4 (36.4%)	0.07
Female	13 (92.9%)	7 (63.6%)	
Age	33 (18–49)	23 (18–62)	0.443^a^
CARE score			
1	6 (42.9%)	3 (27.3%)	
2	4 (28.6%)	1 (9.1%)	1.000
3	4 (28.6%)	7 (63.6%)	
NYHA scale			
I	4 (28.6%)	6 (54.5%)	
II	9 (64.3%)	3 (27.3%)	0.192
III	1 (7.1%)	2 (18.2%)	1.000
IV	–	–	
PH	8 (57.1%)	6 (54.5%)	1.000

^a^) Presented as median (range) with Mann-Whitney U test

## Discussion

In the last decade, the management of the congenital cardiac malformation has been rapidly improved in developed countries, even a very complex lesion can now be treated. However, it is the opposite for developing countries with limited health resources. Many pediatric patients with congenital heart disease do not have access to adequate healthcare. This is a great challenge for practitioners [3].

In the developed countries, the diagnosis of CHD is established at the age of 1 week after birth in 40–50% of patients and in 1 month in 60%. Although in some developed countries prenatal diagnosis has been used to detect most of the CHD before birth, in developing countries only a small proportion of children with CHD may be detected. Due to delays, lack of skilled personnel and surgical facilities in the previous level of referral hospital, most of patients were diagnosed after advanced stages, and therefore surgical treatment was not indicated. The diagnosis of CHD in developing countries are often delayed, when complications such as heart failure, pulmonary vascular obstructive disease, severe cyanosis and infective endocarditis already develop [1].

We performed a simple retrospective study to describe the population of congenital heart disease patients who underwent cardiac surgery at Dr. Sardjito Hospital Yogyakarta Indonesia between April 2018 to March 2019. The results showed that most of these patients had ASD lesions. No patients with complex lesions were found in our institution during the study period. A meta-analysis of 260 studies with a total of 130,758,851 live births suggested increased global prevalence of CHD up to 10% every 5 years between 1970 and 2017, where more than 90% of increase was probably due to improved detection of mild heart lesions (VSD, ASD, and PDA). The study found that ASD had increased 6-fold in the period of 2010–2017 compared to the period of 1970–1975. More prevalent use of echocardiography globally, and improved echocardiographic techniques may explain the increased prevalence of mild lesions. However, the study also reported heterogeneity in the change of prevalence of CHD in different regions, with Asia showing the most marked rise in prevalence of mild lesions. This may be due to drastic improvement in the socioeconomic status and better access to healthcare or potentially higher exposure to genetic or environmental factors such as air particulate matter (PM) pollution in lower-income countries [4]. PM is already known as indpendent risk factor for cardiovascular morbidity and mortality [5]. PM exposure also increased the risk of congenital cardiac defects, especially during the first 3 months of pregnancy [6].

It should be noted that indication of surgery in the current study were because of the complications of their disease. Most of the patients had either left to right shunt and/or pulmonary hypertension. Majority of patients had only defect closure surgery, with exception of three patients. One patient had ASD closure along with mitral valve repair and de Vega procedure. This patient were diagnosed with ASD II, mild pulmonary hypertension, and moderate mitral regurgitation. VSD closure with infundibular resection and thrombus evacuation was performed in another patient diagnosed with peri-membranous VSD, infundibular pulmonary stenosis, septic pulmonary embolism and infective endocarditis. And finally, a VSD closure with intra-right ventricle septal resection was performed in a patient with VSD with double chamber right ventricle. ASD closure is indicated in the presence of any hemodynamically significant shunt causing enlargement of right heart structures, irrespective of the presence of symptoms. Other indications for ASD closure include the rare cases of documented orthodeoxia-platypnea—regardless of shunt size, and confirmed paradoxical embolism [7]. Meanwhile, common indications for VSD closure included aortic regurgitation, pulmonary hypertension, and associated defects [8].

Mean age of patients with congenital heart disease during cardiac surgery in our study was 31.15 ± 13.63 years. The number of adolescents and adults with diverse CHD may reflect the lack of pediatric cardiologist and local surgical facilities. These patients usually had simple congenital heart defects, undetectable, or complex cyanotic malformations that may still be compensated that enables them to achieve a good quality of life until complications appear due to aging [1].

We also found that most of the CHD patients who underwent surgery in our center were female. The role of gender in CHD outcomes has been largely ignored. The predominance of female in our study may be explained by purely coincidence due to small sample size, or there is biological and developmental explanation for this. A large study of European population of adult CHD patients found that a larger proportion of females were symptomatic, yet mortality was higher in males during five year follow up [9]. Another study found that women were more prone to develop pulmonary hypertension, while men had higher risk of endocarditis and aortic complications [10]. The role of gender in CHD should be explored further.

Clinical presentations of CHD vary depending on the type of defects, severity, and age at diagnosis. In newborns, patients may present with asymptomatic heart murmur, cyanosis, heart failure, or shock. For toddler and younger children, the symptoms may include heart murmur, less food intake, failure to thrive, cyanosis, finger clubbing, hypoxic attacks, and heart failure. In adolescents, the symptoms may include murmur, chest pain, arrhythmia, syncope, hypertension, and heart failure [11].

Our results found that mostly of our patients had pulmonary hypertension. Combined together with type of defects and age at surgery, it is suggested that our patients may had simple lesion at birth but had been progressing and complicated by pulmonary hypertension. Pulmonary hypertension is defined as an increase in mean pulmonary arterial pressure ≥ 25 mmHg during rest [12]. The pulmonary hypertension can be classified on the basis of the underlying mechanisms, or the developmental or acquired anatomic abnormalities [13]. Pulmonary hypertension can be generally classified into: pulmonary arterial hypertension (PAH group I), PH due to left heart disease (group II), PH due to pulmonary disease (III), chronic thromboembolic PH (IV), and idiopathic multifactorial PH (V). CHD patients are often classified into the PAH group I with hemodynamic characteristics of precapillary PH (pulmonary vascular resistance (PVR) > 3 woods units (WU) and pulmonary artery wedge pressure ≤ 15 mmHg) [14].

Patients with congenital heart disease are known to have higher risk for PH. The development of PH has been associated with poor prognosis if not treated, compared to patients with congenital heart disease without pulmonary hypertension. The CHD-related PAH (PAH-CHD) usually develops due to intracardiac or extracardiac shunt without inhibited pressure and/or volume overload in pulmonary circulation. This, in time will induce a shear stress, damage of arterial endothelial, and pulmonary vascular remodeling. Although the prevalence of PAH-CHD is still unknown, it is estimated that around 10% of adults with CHD have PAH, that may affects the quality of life and outcome [15]. Pulmonary hypertension may occur at any point in life of CHD patients, and when develop, will adversely affect the quality of life, physical performance, and morbidity and mortality. Progress in surgical techniques and CHD percutaneous treatment has enabled repair or palliation of complex defects, with a decrease in perioperative mortality. However, the closure of the defect in patients with the increased pulmonary vascular resistance may have a long-term adverse effect, even in patients who responded to PAH therapies. Patients with residual PAH after “successful” closure of the defect had worse long-term outcome when compared with patients with more severe PAH but with intra- or extracardic communication, as in patients with Eisenmenger syndrome [16].

A number of limitations of this study should be considered. First, this was a retrospective study by extracting data from INACARD database. This database only include in-hospital mortality without long term follow up. Second, no patients had poor outcome during post-operative hospitalization, which may be affected by using in-hospital mortality as outcome and not considering 30 days post-operative mortality when the patient has been discharged from the hospital. Third, the study had relatively small sample size. Thus, this study could not assess the possibility of causality relationship between risk factors with outcome.

## Conclusion

Our study suggested that surgical procedures may be performed sucessfully in adult patients with congenital heart disease with low morbidity and mortality. All adult CHD patients in the study showed satisfactory outcomes. All patients were treated in ICU for less than two days. No differences were found in terms of age, gender, lesion types, NYHA class, CARE score, and PH between prolonged and non-prolonged hospital LOS groups. A large proportion of patients had PH. Early detection and timely management are needed to avoid the progression of disease and the development of complications, especially pulmonary hypertension. Further studies are needed using 30-days mortality as outcome, to include death events that may occur after discharge from hospital. Studies with prospective design and longer follow-up may better reflect poor outcomes and risk factors associated with them.

## Data Availability

The data of this study are available at Department of Anesthesiology and Intensive Care, Faculty of Medicine, Public Health, and Nursing, Universitas Gadjah Mada. Qualified researchers may request these data directly through authors or their institution.

## Abbreviations

ACE: Angiotensin converting enzyme
ARB: Angiotensin II receptor blocker
ASD: Atrial septal defect
CARE: Cardiac anesthesia risk evaluation
CHD: Congenital heart disease
CPB: Cardiopulmonary bypass
ICU: Intensive care unit
INACARD: Indonesia cardiovascular anesthesia registry database
LOS: Length of stay
NYHA: New York heart association
PAH: Pulmonary arterial hypertension
PAH-CHD: CHD-related PAH
PH: Pulmonary hypertension
PM: Particulate matter
VSD: Ventricle septal defect
